# Higher Speciality Training Boot Camp in Otolaryngology: A Quantitative and Qualitative Analysis of the Northern National Formative Specialty Training 3 Induction Course

**DOI:** 10.7759/cureus.20308

**Published:** 2021-12-09

**Authors:** Rajesh Anmolsingh, Rohma Abrar, Bhargavi Chandrasekar, Joseph Salem, Rachel Edmitson, Rajeev Advani, Sadie Khwaja, Simon Watmough, Nirmal Kumar

**Affiliations:** 1 School of Surgery, Health Education England North West, Manchester, GBR; 2 Otolaryngology, Manchester University NHS Foundation Trust, Manchester, GBR; 3 ENT, Alder Hey Children’s Hospital, Liverpool, GBR; 4 Otolaryngology, Alder Hey Children’s Hospital, Liverpool, GBR; 5 Medicine, Edge Hill University, Ormskirk, GBR; 6 Otolaryngology, Wrightington, Wigan and Leigh NHS Foundation Trust, Wigan, GBR

**Keywords:** data analysis (quantitative and qualitative), otolaryngology training, postgraduate education, simulation in medical education, boot camps

## Abstract

Background

Opportunities for new otolaryngology trainees to develop their skills as they embark on specialty training can be limited. Our facility hosted a national simulation-based boot camp for new otolaryngology trainees in the UK. This study aimed to assess the effectiveness of the boot camp in improving trainee confidence as they transitioned from core surgical training (CST) to higher specialty training (HST) in otolaryngology.

Methodology

We conducted a prospective study on the effectiveness of the boot camp on trainee induction. The boot camp included hands-on simulation, small group teaching and didactic lectures addressing technical skills in the fields of otology, laryngology, rhinology, facial plastics, and paediatrics, as well as non-technical skills involving human factors, simulated ward round, and cognitive simulation. The boot camp curriculum reflected the competencies expected by the Joint Committee of Surgical Training (JCST) at this level of training. Participants completed a pre- and post-course questionnaire addressing their self-confidence for the technical and non-technical skills they developed during the boot camp. All participants were invited to participate in an interview 12 months after the boot camp.

Results

A total of 27 new otolaryngology trainees (approximately half of all new otolaryngology trainees in the UK) participated in the boot camp. A significant increase in median confidence was observed for all technical and non-technical stations (p < 0.0001). The increase in confidence observed was similar for participants regardless of prior experience in otolaryngology. Five candidates were interviewed a year after the boot camp. Analysis of the transcripts generated distinct comments that were grouped into five key themes.

Conclusions

A simulation-based boot camp mapped to the JCST curriculum can increase the confidence of new otolaryngology Specialty Training 3 trainees during their transition from CST to HST. It can provide valuable and durable technical and non-technical skills to aid trainees in the clinic, theatre environment, and when on-call during their inaugural year of training.

## Introduction

Otolaryngology training pathway in the UK

The otolaryngology training pathway in the UK is a six-year programme. Following the completion of a two-year Core Surgical Training (CST) programme, trainees are recruited through a highly competitive national selection process to embark on the otolaryngology Specialty Training (ST) programme. Trainees are allocated the title ST3 on entry to higher surgical training (HST) and complete training at the completion of ST8 [1].

At each stage of surgical training, trainees are expected to attain specific surgical and clinical competencies defined by the Joint Committee of Surgical Training (JCST) [2]. JCST develops and maintains standards across all surgical training programmes in the UK, within the General Medical Council’s (GMC) regulatory framework. The end of ST is awarded by a ‘certificate of completion of training’ (CCT) that requires completion of intercollegiate and fellowship examinations as well as completion of surgical training competency-based assessments and logbook evidence, as outlined by the JCST.

Role of simulation-based boot camp for induction training

Approximately 20 to 50 new HSTs enter otolaryngology specialty training in the UK every year [3]. New ST3 trainees are expected to improve their confidence and competence as they progress through training via traditional means of surgical education that revolves around the apprenticeship model of observation, coaching, and practice supplemented with lecture-based departmental teaching and paid courses [4]. Simulation-based education has been increasingly utilised in surgical education over the past few years as it offers a unique opportunity to improve surgical technical skills, knowledge, and confidence in trainees, and can lead to better patient health outcomes without being inherently reliant on using real patients to deliver the training [5-7]. Actively engaging in deliberate practice of tasks, as in simulation-based training, with immediate feedback from tutors and peers has been shown to better develop professional expertise in trainees [8].

Surgical boot camps to deliver simulation-based education are increasing in popularity in the UK [9-11]. They are designed to be intensive, highly focused courses that integrate core theoretical and practical methods allowing participants to gain new skills and knowledge effectively and efficiently both in a short time period and in a safe learning environment. Simulation-based boot camps can help trainees develop their management skills for uncommon but critical events that trainees may otherwise not get exposed to until they are faced with a real-life emergency scenario for the first time [5,6,12,13]. Although the effectiveness of surgical boot camps has previously been assessed by pre- and post-course evaluation [10,11,14], less is known about the durability and relevance of such learning once the new surgical trainees have gained exposure during their first year of speciality training.

Formation of the ST3 otolaryngology boot camp

Following the success of the junior doctors’ ENT emergency training course by Swords et al., which aimed at improving confidence and skill competency for new on-call otolaryngology doctors [13], we hypothesised that an ST3 induction boot camp would enhance the experience of CSTs and increase their confidence, professional practice, and performance as they commence otolaryngology ST. We sought to evaluate the impact on their first year of HST.

## Materials and methods

Participants

New otolaryngology ST3 trainees were recruited by national advertisement on the ENT UK website. ENT UK is the national representative body of otolaryngology surgeons.

Course design

A course curriculum was designed to reflect the ST3 competencies expected as per the JSCT guidelines. Over the two-day intensive course, regional consultants and senior specialty trainees led a total of eight technical and non-technical/human factors stations. Each station was 30 minutes long and was mapped to the course curriculum (Table 1).

**Table 1:** Technical and non-technical stations.

Stations	Skills covered
Technical stations	
Otology	Setting up a microscope and facial nerve monitor for mastoidectomy; assessing temporal bone anatomy and performing cortical mastoidectomy using a temporal bone simulator (Voxelman simulator)
Laryngology	Performing microlaryngoscopy for vocal cord surgery; performing tracheostomy; performing rigid oesophagoscopy; performing fine-needle aspiration
Rhinology	Assessing anatomy and pathology using fibreoptic endoscopic sinus surgery; controlling epistaxis
Facial plastics	Wound closure using skin flaps; suture techniques on animal cadaver skin; inserting and monitoring drains
Paediatrics	Managing paediatric airway (high-fidelity interactive mannequin); removal of foreign body; managing blocked tracheostomy; performing bronchoscopy
Non-technical stations	
Human factors	Human error; peri-operative planning; communication with staff members (used Sim-man – Laerdal Medical)
Simulated ward round	Leading and managing a ward round; decision-making; communication with a multidisciplinary team
Cognitive simulation	Application of cognitive simulation to surgical training

**Figure 1:**
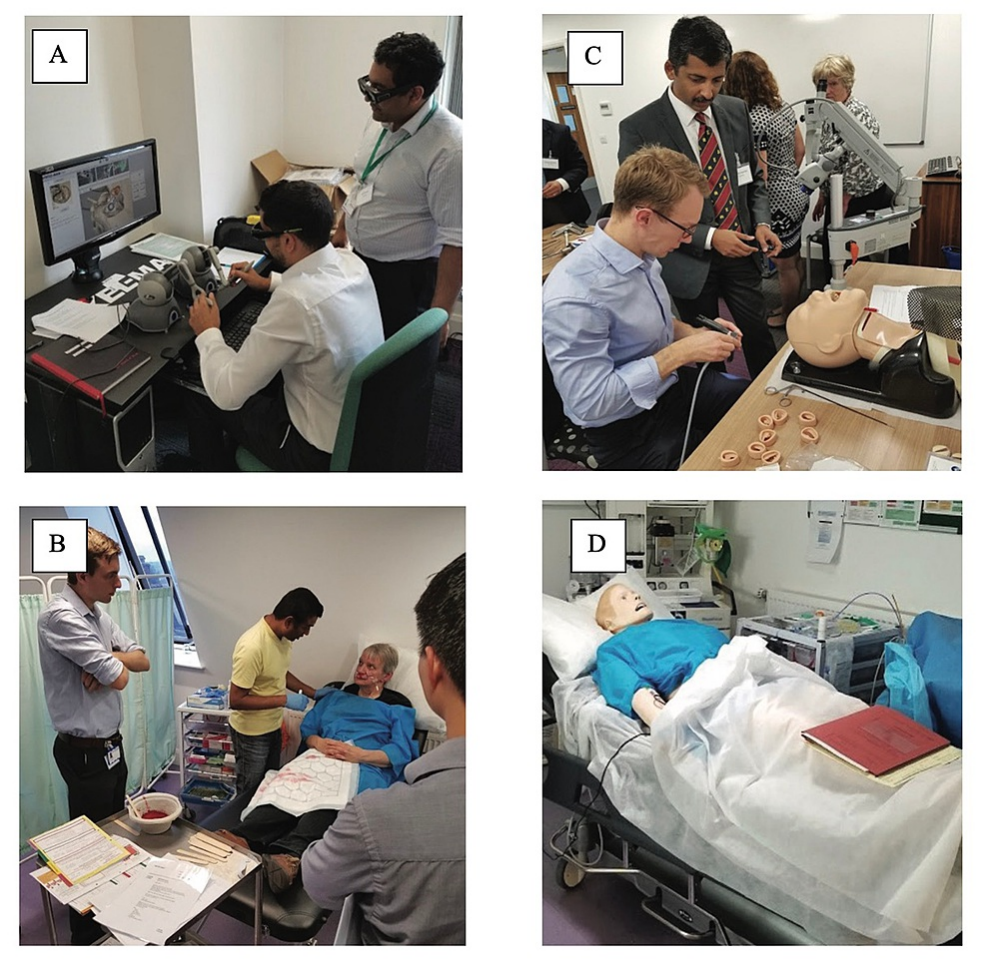
Technical and non-technical simulation scenarios. (A) A participant attempting cortical mastoidectomy using a Voxel-Man temporal bone simulator. (B) A simulated ward round where a participant leads a team of juniors in the assessment and management of a patient with epistaxis. (C) A participant setting up a laryngoscope to perform microlaryngoscopy. (D) Interactive high-fidelity SimMan^TM^ mannequin (Laerdal Medical, Stavanger, Norway) used for acute airway emergency station.

Technical and non-technical stations employed actors, high-fidelity mannequins, and cadaveric models to enhance the immersive simulation experience of participants (Figure 1). The skills gained in each station were reinforced through didactic lectures throughout the course. Real-time feedback was provided by the consultants during each station as well as at the end of each scenario with informal assessments mapped to the JCST curriculum to guide candidates.

Study design

A prospective, single-blinded study was performed to assess the impact of the boot camp using quantitative pre- and post-course evaluation. Qualitative interviews were conducted to assess the effectiveness and clinical impact of the course 12 months after completion.

Independent academic approval to carry out this study was granted by the local Director of Medical Education and the Research and Development team at Wrightington, Wigan and Leigh Foundation Trust.

Data collection

Feedback questionnaires were provided to participants both at the start and end of the course. Participants were asked to rate their confidence level for each station using a seven-point Likert scale (one - strongly disagree, seven - strongly agree). Confidence levels were assessed using two questions of each station. These confidence levels were summed to give an overall confidence rating out of 14 for each technical and non-technical station.

All participants were invited to participate in an interview 12 months after the boot camp. These voluntary interviews were conducted in a semi-structured format with open-ended questions, followed by generic probes by a single investigator (RA). Verbal informed consent was obtained during the interview. Audio recordings of the participants were transcribed by a medical secretary. All transcriptions were rendered anonymous during this transcription process.

Data analysis

Course Quantitative Outcomes

Participant confidence ratings pre- and post-course for each station were compared using the Wilcoxon signed-rank test. In addition, the pre-course confidence ratings of participants with ≤18 months of prior experience in ENT compared to those with >18 months were compared using the Mann-Whitney test. Comparisons between participants with greater than and less than 18 months of prior experience in ENT were also made with this cut-off used to penalise otolaryngology hopefuls at ST3 applications by Health Education England [15].

Data were analysed using Prism 7.0d software (GraphPad Software, San Diego, California, USA) and SPSS Statistics for Macintosh, version 1.0.0.1347 (IBM Corp., Armonk, NY, USA).

Course Qualitative Outcomes

Grounded theory, a fundamental qualitative descriptive approach, was used to analyse the interview transcripts. Independent analysis of participant statements was undertaken to generate initial codes through careful interpretation of the transcripts. Several iterative cycles were used to develop preliminary codes using a constant comparison technique, and emerging categories from the codes were refined to identify core themes. All qualitative analysis was undertaken using Dedoose Version 8.3.17 web software.

Ethical approval

Ethical approval was not required for this study as advised by The National Research Ethics Service.

## Results

The study recruited 27 doctors to the boot camp. This cohort represented half the number of all new ST3 doctors in the UK.

Quantitative analysis of pre- and post-course confidence levels

All 27 participants completed the pre- and post-course questionnaire. Candidate confidence level pre-course was highest for laryngology (median confidence rating of 11), followed by human factors and simulated ward round station (median confidence ratings of 10). Candidates rated themselves the least confident pre-course for otology, paediatrics, and cognitive simulation (all had a median confidence rating of 7).

Comparison of Pre- and Post-Course Confidence Levels

**Figure 2:**
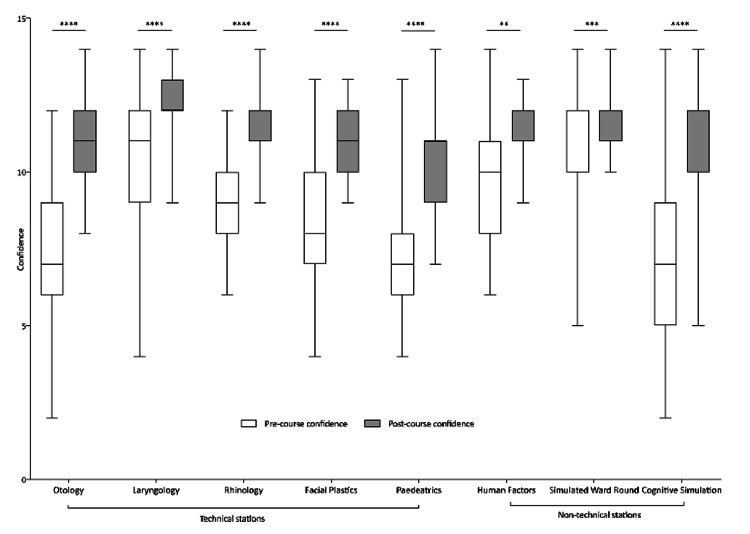
Box and whisker plot demonstrating significant increase in participant confidence ratings from pre-course to post-course for all technical stations and non-technical stations using the Wilcoxon signed-rank test (**p < 0.005, ***p < 0.0005, ****p < 0.0001).

There was a significant increase in confidence ratings of all candidates’ post-course for each station with an overall increase of median confidence level from 9 to 12 out of 14 (p < 0.0001) (Figure 2).

The largest increase in confidence was observed in cognitive simulation (median confidence increase of 5 points, (p < 0.0001). This was followed closely by otology and paediatrics, both of which had a median confidence increase of 4 points (p < 0.0001). Laryngology and human factors stations showed the smallest increase in confidence ratings [mean confidence increase of 1 point; laryngology (p < 0.0001), human factors (p < 0.005)].

Relationship of Previous Experience in ENT With Pre- and Post-Course Confidence

Participants with greater previous experience in ENT (>18 months) had higher pre-course confidence for otology compared to those with ≤18 months experience (p < 0.05). However, no such significant difference was observed for all other technical and non-technical stations. No significant difference was observed in the percentage increase from pre- to post-course confidence for all technical and non-technical stations when comparing participants with prior experience of ≤18 and >18 months in ENT.

Thematic analysis of post-course interviews

In total, five of the 27 attendees of the Northern ST3 Induction Boot Camp participated in the post-course interview 12 months after the initial boot camp.

Interview transcripts were anonymised by ascribing each interviewee a unique identification label from P1-5. Analysis of these transcripts generated distinct comments that were mapped to 22 distinct codes and subsequently grouped into five major themes: (1) content and design of the stations; (2) impact on the transition from CST to ST3 in ENT; (3) development of technical and non-technical skills; (4) impact on clinical performance in ST3; and (5) recommendations for future boot camps.

Theme 1: Content and Design of the Stations

Participants felt that the curriculum for the boot camp was comprehensive and was delivered in a realistic simulated manner when compared to similar scenarios the participants had subsequently encountered during their first year of surgical training (Table 2).

**Table 2:** Theme 1 – content and design of stations. n = number of distinct participant statements mapped to each code.

Codes	n	Representative participant statements
Comprehensiveness	2	“I cannot think of anything that I would take away or add to it” P4
Realism	7	“I think that the station on grommets was very realistic. There was a lot of distraction with the anaesthetist playing music and you were thrown into it, as you are at work. It is very easy to go in and do a grommet on the wrong side” P4
Relevance	10	“We covered a lot of things which were relevant to what we would face as ST3s” P4
Usefulness	11	“I think that overall I found the boot camp incredibly beneficial” P2
Enjoyment	4	“I definitely enjoyed the boot camp” (P5)
Role of simulation	10	“I thought that the (Voxelman) simulator was very good for building up my skills in cortical mastoidectomies.” (P1)

Theme 2: Impact on the Transition From CST to ST3 in ENT

The majority of trainees stated that the boot camp increased their confidence at work as a new ST3 trainee and addressed their anxieties and fears related to their transition from CST to ST3 (Table 3). They felt that the skills gained from the boot camp helped them transition to specialty training and become a safer and more responsible ST3 trainee. Ten individual statements were coded directly as impacting trainee transition from CST to ST training.

**Table 3:** Theme 2 – impact on the transition from CST to otolaryngology ST3 training. n = number of distinct participant statements mapped to each code. CST: Core Surgical Training; ST3: Specialty Training 3

Codes	n	Representative participant statements
Confidence	10	“It really helped my confidence, especially being a new ST3 going into ENT” (P1)
Competence	3	“I think that the boot camp was somewhat useful in making me more competent at an ST3 level” (P5)
Responsibility	2	“The boot camp made me feel more responsible” (P5)
Safety	10	“I felt that after the course I was more confident. I also felt safer which helped with my clinics and theatre performance” (P1)
Anxiety	3	“The boot camp was useful in addressing my anxieties more than anything, mostly about decision-making but also regarding my clinical skill and generally stepping up” (P5)

Theme 3: Development of Technical and Non-Technical Skills

Participants reported the boot camp helped enhance their technical and non-technical skills. Specific new technical skills that they were exposed to for the first time by the boot camp were highlighted. These included facial plastics, functional endoscopic sinus surgery (FESS), paediatric bronchoscopy, and assessment of temporal anatomy. One participant felt that these increased their “learning of different sub-specialties such as rhinology, head & neck and paediatrics” (P1). They also felt that the non-technical stations had been incredibly useful as they had noted the impact of non-technical factors on clinical outcomes during ST3 (Table 4).

**Table 4:** Theme 3 – development of technical and non-technical skills. n = number of distinct participant statements mapped to each code.

Codes	n	Representative participant statements
Exposure to new skills	10	“I think that paediatric bronchoscopy is something that not a lot of people are exposed to and it was useful to get a lot of information on this topic” (P3)
Reinforcement of skills	6	“I think the course filled in some gaps and was a good reflection on what I have done so far and what I will be doing as a registrar” (P2)
Enhancement of technical skills	13	“I thought the simulator was very good for building up my skills in cortical mastoidectomies” (P1)
Enhancement of non-technical skills	11	“There have been a number of occasions where I have felt that non-technical performance has affected the conduct of operations … Discussing and looking at your own mindset and behaviour in the boot camp was very useful” (P2)

Theme 4: Impact on Clinical Performance in ST3

Candidates felt that the technical and non-technical skills had a direct impact on their clinic and theatre performance during their subsequent ST3 year (Table 5). They also felt that they were better equipped to deal with on-call emergencies after the simulation sessions in the boot camp.

**Table 5:** Theme 4 – impact on clinical performance in ST3. n = number of distinct participant statements mapped to each code. ST3: Specialty Training 3

Codes	n	Representative participant statements
Clinic performance	4	“I felt safer after the course which helped with my clinics and theatre performance” (P1)
Theatre performance	18	“The communication station and theatre scenarios come up whilst you are working as an ST3. I think that the boot camp does help you remember what you learnt when you are put into these situations” (P3)
On-call emergencies	11	“Having more information on tracheostomy tubes and speech valves gave me much more confidence especially when working on-call” (P3)

Theme 5: Recommendations for Future Boot Camps

All candidates felt the boot camp would be of benefit to new otolaryngology trainees (Table 6). Suggestions for improvement included ideas to include lectures addressing intra- and post-operative complications as well as simulations set in a clinic setting to practise common clinic scenarios for ST3.

**Table 6:** Theme 5 – future recommendations. n = number of distinct participant statements mapped to each code. CST: Core Surgical Training; ST3: Specialty Training 3

Codes	n	Representative participant statements
Recommendations to colleagues	3	“I would recommend the boot camp to upcoming CST2s” (P3)
Making the course mandatory	6	"I think that the boot camp should be mandatory for all trainees becoming ST3s” (P5)
Recommendations of new topics for subsequent boot camps	3	“I know that clinics are relatively new to a person starting as an ST3 and I think that one or two lectures on how consultants will expect us to deal with common scenarios in clinics that we are faced with would be very helpful” (P1)
Recommendations for other specialties	5	“I think it would be a very good idea for every specialty to have these types of courses” (P1)

## Discussion

To our knowledge, this study is the first to assess the benefits and relevance of an ENT induction boot camp for new higher surgical trainees in the UK immediately after and one year following a boot camp using quantitative and qualitative methods, respectively.

We found that an ST3 induction boot camp is effective in achieving a statistically significant improvement in the confidence levels of new otolaryngology trainees for technical and non-technical skills expected by the JCST at the ST3 level. We observed the greatest increase in confidence for cognitive simulation, otology and paediatrics stations. The significantly lower pre-course confidence for these stations and the subsequent greatest increase from pre- to post-confidence is likely secondary to the lack of exposure to these skills during CST. For example, paediatric airway training only occurs at specialised tertiary centres within the UK and is not included in the CST curriculum, as highlighted in the participant comments that the boot camp taught them skills such as paediatric bronchoscopy. The observed increase in confidence levels suggests the inductive boot camp offered an effective introduction to these specialist areas and addressed the deficiencies in trainees’ knowledge.

In contrast, we observed the smallest increase in confidence for laryngology and human factors stations. Trainees have greater exposure to laryngology during CST, and therefore, it is unsurprising that trainees had a higher pre-course confidence level in this area. Despite this, we observed a small but statistically significant increase in confidence levels. This suggests that such stations provide a useful opportunity to revisit and consolidate pre-existing knowledge and skills.

The boot camp aimed to accelerate learning by creating high-fidelity stations using computer-aided simulations and mannequins in the technical stations, as well as the use of actors to role play theatre personnel in the non-technical stations, to achieve high equipment, environment, and psychological fidelity. High-fidelity simulations have been shown to be useful for practising technical skills and management of specific intra-operative scenarios [16]. The value of high-fidelity simulations is reinforced by effectively recreating a surgical operating environment while being financially and logistically feasible.

By conducting interviews one year following the boot camp, we were able to assess the effect the boot camp had on trainees’ clinical practice. Our qualitative analysis found that the boot camp had a positive impact on trainees’ transition from CST and ENT, as trainees reported a direct impact on clinical, theatre, and on-call performance.

Despite a small increase in confidence levels immediately following the boot camp, participants reported a year on that they found the non-technical or human factors stations useful and had experienced first-hand how important such skills were in theatre during ST3 training, suggesting it still has a positive impact on clinical practice. This is encouraging as poor communication and deficiencies in non-technical skills have been linked to a large proportion of fatal medical accidents, near misses in practice, and malpractice claims [17,18]. The importance of non-technical skills and surgical safety is now recognised and is reflected by the dissemination of non-technical skills for surgeons courses, first developed by the Royal College of Surgeons of Edinburgh in 2006 [19].

However, there is much debate surrounding the relationship between confidence and skill, with an increase in confidence not necessarily translating into an increase in competence [20]. Some studies in the literature have proposed that confidence levels in practising doctors may not be proportional to their skill set, whereas others have identified self-confidence as being important in acquiring skills and improving performance [21,22]. Other boot camps that have analysed the change in confidence and objective improvement in knowledge or skills have tended to show a similar increase in both parameters [23]. We did not investigate whether the increased level of participant-reported confidence impacted patient outcomes. However, McGaghie et al. reported that simulation-based education can improve patient safety and contribute to patient care [7].

The effectiveness of a surgical boot camp compared to no intervention has been demonstrated in randomised control trials [24,25]. However, there are no studies comparing the effectiveness of boot camps versus other teaching courses. Few studies reviewed the benefit of simulation-based education compared to other traditional medical education in medical students. In this cohort, the benefit of simulation on knowledge and skill acquisition compared to other teaching methods, including case-based discussion and apprenticeship, is inconclusive but has been shown to improve participant confidence [26-28]. However, there is a lack of data on surgical trainees and outcomes from undergraduate education cannot be assumed to reflect postgraduate training.

Limitations

Our main limitation is the lack of knowledge and skill-based assessment before and after the boot camp. This was intentional to encourage participant engagement without fear of judgement by consultants or peers they will encounter in HST. However, it limited our ability to gauge improvement in competence and skill objectively. The study did not have a comparative control group and the sample size for interviews was small. With interviews conducted one year after the boot camp, it is possible that recall bias would have affected the accuracy of the results. Further analysis with a larger sample size at a chosen set point following the course is required to better understand the effects of boot camps on otolaryngology training.

## Conclusions

Our study suggests that an induction boot camp course, mapped to the JCST curriculum and delivered in intensive small group simulation sessions, can increase the confidence of new otolaryngology ST3 trainees in their transition from CST to HST. It can provide valuable and durable technical and non-technical skills to aid trainees in the clinic, theatre environment, and when on-call during their inaugural year of training. The integration of simulation-based induction boot camps into postgraduate training may further support and enhance trainees transitioning from CST to HST in ENT and other surgical specialties within the UK.
